# Safety and tolerability of sorafenib in patients with radioiodine-refractory thyroid cancer

**DOI:** 10.1530/ERC-15-0252

**Published:** 2015-12

**Authors:** Francis Worden, Martin Fassnacht, Yuankai Shi, Tatiana Hadjieva, Françoise Bonichon, Ming Gao, Laura Fugazzola, Yuichi Ando, Yasuhisa Hasegawa, Do Joon Park, Young Kee Shong, Johannes W A Smit, John Chung, Christian Kappeler, Gerold Meinhardt, Martin Schlumberger, Marcia S Brose

**Affiliations:** 1 Division of Hematology/Oncology, University of Michigan Comprehensive Cancer Center, University of Michigan Health System, 1500 E. Medical Center Drive, Ann Arbor, Michigan, 48109, USA; 2 Endocrine Unit, Department of Medicine I, University Hospital, University of Würzburg, Würzburg, Germany; 3 Comprehensive Cancer Center Mainfranken, University of Würzburg, Würzburg, Germany; 4 Department of Medical Oncology, Cancer Hospital, Chinese Academy of Medical Sciences and Peking Union Medical College, Beijing, China; 5 Beijing Key Laboratory of Clinical Study on Anticancer Molecular Targeted Drugs, Beijing, China; 6 Radiotherapy Department, Medical University, Sofia, Bulgaria; 7 Institut Bergonie, Bordeaux, France; 8 Tianjin Medical University Cancer Hospital, Tianjin, China; 9 Fondazione IRCCS Ca' Granda, Milan, Italy; 10 Department of Pathophysiology and Transplantation, University of Milan, Milan, Italy; 11 Nagoya University Hospital, Nagoya, Japan; 12 Aichi Cancer Center Hospital, Nagoya, Japan; 13 Seoul National University College of Medicine, Seoul, Korea; 14 Asan Medicine Center, Seoul, Korea; 15 Department of Internal Medicine, Radboud University Nijmegen Medical Center, Nijmegen, The Netherlands; 16 Bayer HealthCare Pharmaceuticals, Montville, New Jersey, USA; 17 Bayer Pharma AG, Berlin, Germany; 18 Institut Gustave Roussy, Villejuif, France; 19 Department of Otorhinolaryngology, Head and Neck Surgery, Abramson Cancer Center of the University of Pennsylvania, Philadelphia, Pennsylvania, USA

**Keywords:** differentiated thyroid cancer, sorafenib, targeted therapy, tyrosine kinase inhibitor, adverse events

## Abstract

Effective adverse event (AE) management is critical to maintaining patients on anticancer therapies. The DECISION trial was a multicenter, randomized, double-blind, placebo-controlled, Phase 3 trial which investigated sorafenib for treatment of progressive, advanced, or metastatic radioactive iodine-refractory, differentiated thyroid carcinoma. Four hundred and seventeen adult patients were randomized (1:1) to receive oral sorafenib (400 mg, twice daily) or placebo, until progression, unacceptable toxicity, noncompliance, or withdrawal. Progression-free survival, the primary endpoint of DECISION, was reported previously. To elucidate the patterns and management of AEs in sorafenib-treated patients in the DECISION trial, this report describes detailed, by-treatment-cycle analyses of the incidence, prevalence, and severity of hand–foot skin reaction (HFSR), rash/desquamation, hypertension, diarrhea, fatigue, weight loss, increased serum thyroid stimulating hormone, and hypocalcemia, as well as the interventions used to manage these AEs. By-cycle incidence of the above-selected AEs with sorafenib was generally highest in cycle 1 or 2 then decreased. AE prevalence generally increased over cycles 2–6 then stabilized or declined. Among these AEs, only weight loss tended to increase in severity (from grade 1 to 2) over time; severity of HFSR and rash/desquamation declined over time. AEs were mostly grade 1 or 2, and were generally managed with dose interruptions/reductions, and concomitant medications (e.g. antidiarrheals, antihypertensives, dermatologic preparations). Most dose interruptions/reductions occurred in early cycles. In conclusion, AEs with sorafenib in DECISION were typically grade 1 or 2, occurred early during the treatment course, and were manageable over time.

## Introduction

The early identification and proactive management of adverse events (AEs) are fundamental to oncology practice and particularly to the optimal use of newer, targeted anticancer therapies such as sorafenib – an oral multikinase inhibitor of vascular endothelial growth factor receptor (VEGFR)-1, VEGFR-2 and VEGFR-3, RET (including RET/PTC), RAF (including BRAF^V600E^), and platelet-derived growth factor receptor β (Wilhelm *et al*. 2004, Carlomagno *et al*. 2006). Sorafenib has demonstrated a consistent safety profile across tumor types. AEs associated with sorafenib are predominantly grade 1/2, non-life threatening, and manageable. The most commonly reported AEs include hand–foot skin reaction (HFSR), rash/desquamation, hypertension, diarrhea, fatigue, and weight loss (Escudier *et al*. 2007, Llovet *et al*. 2008, Cheng *et al*. 2009, Brose *et al*. 2014*a*).

DECISION was a large Phase 3 randomized, placebo-controlled trial in patients with locally advanced or metastatic radioactive iodine (RAI)-refractory differentiated thyroid cancer (DTC); sorafenib significantly improved progression-free survival (PFS) vs placebo (hazard ratio 0.59; 95% CI 0.45–0.76; *P*<0.0001; median PFS 10.8 vs 5.8 months respectively) (Brose *et al*. 2014*a*). Given the long duration of sorafenib therapy that was observed in this trial (median 10.6 months; interquartile range 5.3–15.7), it is particularly important to understand its safety profile in this setting. DECISION, as the first large-scale trial in patients with RAI-refractory DTC, may provide insight into the management of patients receiving sorafenib in this setting.

Here we report a detailed, by-cycle analysis of the incidence, prevalence, and severity of the most commonly reported and clinically relevant treatment-emergent AEs, as well as the associated dose modifications in patients treated with sorafenib in the DECISION trial. We also consider the interventions used to manage these AEs.

## Materials and methods

### Study design

DECISION was a multicenter, randomized, double-blind, placebo-controlled, Phase 3 trial that was conducted in 18 countries in Europe, Asia, and North America. Study details have been previously reported (Brose *et al*. 2011, 2014*a*). Briefly, patients with locally advanced or metastatic RAI-refractory DTC (papillary, follicular (including Hürthle cell), or poorly differentiated) could be enrolled if their disease had progressed within the past 14 months and they had at least one measurable lesion by computed tomography or magnetic resonance imaging. Disease progression and measurable tumors were defined according to Response Evaluation Criteria in Solid Tumors (RECIST v1.0) (Therasse *et al*. 2000). Patients were required to be aged ≥18 years; have an Eastern Cooperative Oncology Group (ECOG) performance status of 0–2; adequate bone marrow, liver, and renal function; and serum thyroid stimulating hormone (TSH) <0.5 mIU/l.

Patients were randomized (1:1) to receive either sorafenib 400 mg (2×200 mg tablets) twice daily or matching placebo. Treatment was continued until disease progression, unacceptable toxicity, noncompliance, or withdrawal of consent. Treatment cycle length was 28 days. Patients all gave written informed consent. Trial conduct and patient safety were monitored by an independent data monitoring committee (Brose *et al*. 2014*a*). The conduct of this clinical study met all local legal and regulatory requirements. The study was conducted in accordance with the ethical principles originating in the Declaration of Helsinki and the International Conference on Harmonization (ICH) guideline E6: Good Clinical Practice.

### Analysis of dose modifications by treatment cycle

Study drug interruption (and reintroduction), dose reduction (and re-escalation), and permanent discontinuation were employed based on protocol-defined criteria, which differed for hematologic AEs, skin toxicities, hypertension, and any other AEs (Supplementary Tables 1, 2, 3 and 4, see section on supplementary data given at the end of this article) (Brose *et al*. 2014*a*). Dose levels were 800 mg (starting dose), 600 mg (divided doses: 400 and 200 mg), 400 mg (divided: 2×200 mg), and 200 mg per day. After dose reductions, the protocol allowed re-escalation upon resolution of the AE.

Dose modifications and treatment discontinuations due to AEs were recorded by treatment cycle. Dose reduction during a treatment cycle was defined as patients receiving at least one daily dose of <800 mg during that cycle.

### Analysis of common AEs by treatment cycle

Patients were assessed for safety every 28 days (i.e. once every cycle) for the first nine treatment cycles, and then every 56 days thereafter for the duration of treatment and 30 days after the last dose. The detailed analyses per cycle reported here are limited to treatment cycles 1–9.

The overall incidence of AEs was reported previously (Brose *et al*. 2014*a*). The by-cycle incidence, prevalence, and severity of the following AEs were assessed in detail: HFSR, rash/desquamation, hypertension, diarrhea, fatigue, weight loss, increased serum TSH, and hypocalcemia. With the exception of HFSR and elevated TSH, all other AEs were reported and graded using the National Cancer Institute Common Terminology Criteria for AEs (CTCAE) v3.0 and Medical Dictionary for Regulatory Activities (MedDRA) v15.1 terminology (National Cancer Institute 2006, International Conference on Harmonisation 2006). The severity of HFSR was assessed using study-specific grading definitions (Supplementary Table 5, see section on supplementary data given at the end of this article); example photographs illustrating the appearance of different HFSR grades in sorafenib-treated patients are shown in Supplementary Figure 1 (see section on supplementary data given at the end of this article) (Chu *et al*. 2008). Elevated TSH (>0.5 mIU/l requiring an increase in the dose of thyroxine replacement) was a study-specific AE, with a maximum severity of grade 1.

The by-cycle incidence of an AE was defined as the number of patients with that AE starting or worsening in a particular cycle. The prevalence of an AE during a treatment cycle was defined as the number of patients with an AE occurring (new or continuing) during that treatment cycle. Both incidence and prevalence are expressed as a percentage of patients at risk in that cycle.

## Results

### Patients

The intention-to-treat population consisted of 417 patients of whom 207 were randomized to sorafenib and 210 to placebo. Patient demographics and baseline clinical characteristics were generally well balanced between the treatment groups (Brose *et al*. 2014*a*). Median age was 63 years and performance status was principally ECOG 0 (62%) or 1 (34%).

### Safety findings for the entire treatment period

#### Overall AE incidence and dose modifications

Safety outcomes across the entire DECISION study treatment period have been reported previously; an overview of these data is shown in Tables 1 and 2 (Brose *et al*. 2014*a*). Overall, the most common AEs in the sorafenib arm were HFSR, diarrhea, alopecia, rash/desquamation, fatigue, weight loss, and hypertension (Table 2). The most common serious AEs (i.e. those reported by ≥2% of patients receiving sorafenib) were dyspnea, pleural effusion, and secondary malignancy (principally squamous cell carcinoma of the skin; *n*=7) (Table 1). Of the specific AEs analyzed in detail in this report, those in the sorafenib group considered to be serious events were fatigue (three patients), weight loss and rash/desquamation (two patients each), as well as HFSR, diarrhea, and hypocalcemia (one patient each). The single patient who experienced serious grade 4 hypocalcemia was hospitalized but recovered with calcium substitution. No case of serious hypertension was reported in this study. There were 12 deaths in the sorafenib group (median time on study 130.5 days) and six in the placebo arm (median time on study 73 days), most of which were attributed to disease progression (seven in the sorafenib group, four in the placebo group; Table 1). One death in each group was attributed to the study drug. One patient receiving sorafenib died of a myocardial infarction 427 days (14.0 months) after starting treatment and one patient receiving placebo died of a subdural hematoma 289 days (9.5 months) after starting treatment (Brose *et al*. 2014*a*).

Study drug interruptions, dose reductions, and permanent discontinuations due to the specific AEs analyzed in this report, and occurring at any time over the course of treatment, are shown in Table 3. In the sorafenib group, HFSR was the most common reason for treatment interruption (in 26.6% of patients) and dose reduction (33.8%). Diarrhea was the next most common reason for dose reduction (13.5%). Overall, permanent discontinuation of the study drug due to AEs occurred in 18.8% of patients in the sorafenib group and 3.8% in the placebo group (Table 1); 11 patients (5.3%) discontinued sorafenib treatment due to HFSR, whereas discontinuations due to other AEs were <1.5% (Table 3).

Concomitant medications could also be used to manage AEs during the DECISION study, either alongside or independently of the study drug dose modifications. The patient records for new concomitant medications introduced over the course of the study showed that, for example, dermatologic preparations were used more frequently in sorafenib patients than in placebo patients. These preparations included corticosteroids (used in 37% vs 19% of sorafenib vs placebo patients respectively) and emollients (34% vs 8%). Reasons for administering concomitant medications were not captured, but it is likely that these were employed to manage dermatologic AEs. The same pattern was evident in use of antidiarrheal medications (61% vs 17%) and antihypertensive medications such as agents acting on the renin–angiotensin system (22% vs 5%) or calcium channel blockers (15% vs 4%). These data, combined with findings from an analysis of per patient data for treatment modifications and AE reporting (Supplementary Figure 2, see section on supplementary data given at the end of this article), suggest that dose modifications in combination with other supportive measures appeared to be effective at reducing AE severity.

### Safety findings by treatment cycle

#### Dose modifications or discontinuations due to AEs by treatment cycle

**Figure 1:**
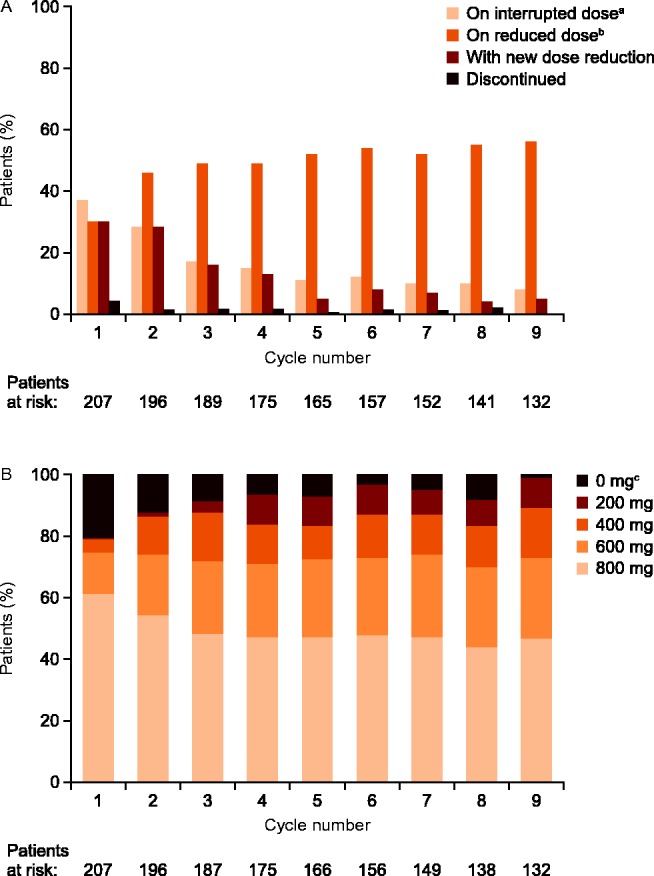
Patients with dose modifications and treatment discontinuations due to AEs in each 28-day cycle of sorafenib treatment (intention-to-treat population). Percentages were calculated using the patients at risk in each cycle as the denominator. (A) Patients on interrupted or reduced doses^a,b^, or with new dose reductions or permanent discontinuations in each cycle. (B) Patients at each dose level at the end of each cycle. ^a^Patients on interrupted dose were defined as those who during the treatment cycle had a new interruption or an interruption continuing from the previous cycle. ^b^Patients on reduced dose were defined as those receiving at least one daily dose of <800 mg during the treatment cycle. ^c^Patients on 0 mg dose at the end of the cycle includes patients who discontinued study drug during the cycle in addition to patients on a dose interruption.

When analyzed by treatment cycle, the proportion of patients with a new or continuing dose interruption in the sorafenib group was highest in cycles 1 and 2 (37 and 28% of patients respectively) and decreased thereafter (8–12% of patients in cycles 5–9; Fig. 1A). The percentage of patients with a new sorafenib dose reduction followed a similar pattern: in cycles 1 and 2, about 30% of these patients had a new dose reduction in cycles 1 and 2; this subsequently declined over cycles 3–5 and was 4–8% of patients during cycles 5–9 (Fig. 1A). The proportion of patients with a new dose reduction, or continuing on a reduced dose implemented in a previous treatment cycle was 30% in cycle 1, increasing in subsequent cycles. It plateaued at ∼49–56% by cycle 3 (Fig. 1A). Discontinuations due to AEs were highest in cycle 1 at 4%, and then occurred at a rate of ∼1–2% in most subsequent cycles (Fig. 1A). The proportion of patients who were receiving either the standard dose (800 mg daily) or the next lower dose (600 mg daily) on the final day in each cycle was relatively stable (∼70%) across cycles 1–9 (Fig. 1B).

Dose modifications were also reported in the placebo group (Table 1). The rate of dose interruption was generally consistent over cycles 1–9, with a range of 4–12%. The rate of new dose reductions in this group was 11% in cycle 1, then around 1–3% in cycles 2–9. The overall rate of permanent discontinuation on placebo was 3.8%; 0–1% over cycles 1–5, then 0% in cycles 6–9.

#### By-cycle incidence and prevalence of common AEs

**Figure 2:**
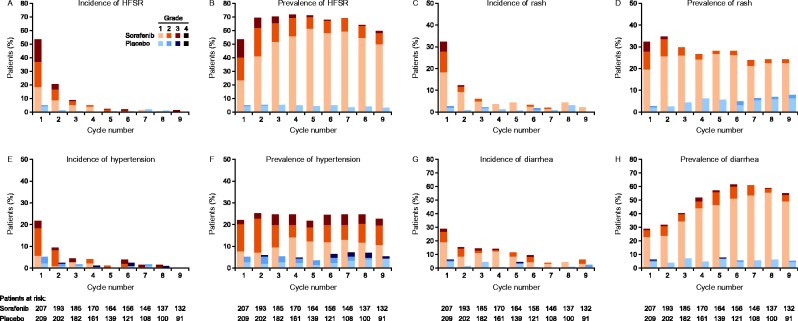
Incidence (onset or worsening) and prevalence (onset or persistence) per 28-day cycle, respectively, for hand–foot skin reaction (HFSR) (A and B), rash/desquamation (C and D), hypertension (E and F), and diarrhea (G and H) during the double-blind treatment period (safety population).

**Figure 3:**
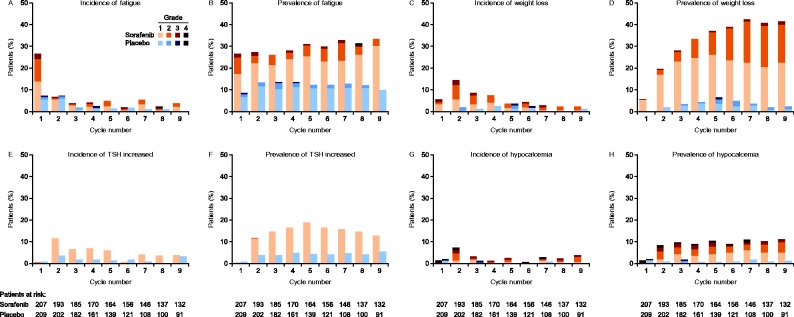
Incidence (onset or worsening) and prevalence (onset or persistence) per 28-day cycle, respectively, for fatigue (A and B), weight loss (C and D), elevated TSH (E and F), and hypocalcemia (G and H) during the double-blind treatment period (safety population). Increased serum TSH was a study-specific adverse event for which grade 1 was the maximum defined severity. Reports of grade 2 increased TSH were due to errors in grading.

The by-cycle incidence of AEs in the sorafenib group was generally highest in cycle 1 or 2, decreasing subsequently. The prevalence of AEs in patients treated with sorafenib tended to increase over the first two to six cycles before stabilizing or declining. In the placebo arm, no general patterns were evident in incidence or prevalence of any of the selected AEs over the first nine cycles (Figs 2 and 3).

In the sorafenib group, the by-cycle incidence of HFSR and rash/desquamation was highest in cycle 1, affecting 54 and 32% of patients at risk in that cycle respectively. The incidence of these AEs decreased exponentially, by approximately half in each subsequent cycle, until cycle 4, after which incidence stabilized. The prevalence of these AEs was generally consistent throughout the first nine cycles, with rates of ∼40–60% for HFSR and of 20–30% for rash. Over time, there was a shift in the severity of HFSR and rash towards lower grades. The proportion of grade 2 and 3 HFSR was highest in cycle 1 and decreased over the first five cycles with a concurrent increase in grade 1. The proportion of grade 2 and 3 rash/desquamation followed a similar pattern, but decreased more quickly, reaching a plateau after cycle 3 (Fig. 2A, B, C and D).

In patients receiving sorafenib, the incidence of hypertension was highest in cycles 1 and 2. Hypertension prevalence remained stable over cycles 1–9 at 22–25%. The severity of hypertension in these patients was generally consistent over time; for example, the prevalence of grade 3 hypertension was within 2–5% throughout cycles 1–9 (Fig. 2E and F).

In the sorafenib group, the by-cycle incidence of diarrhea was highest in cycle 1 at 29%; new onset or worsening of existing diarrhea was reported in 10–15% of patients in each of cycles 2–6. Diarrhea was primarily grade 1 throughout the first nine cycles. Its prevalence increased steadily over the first six cycles, peaking in cycle 6. This increase in overall prevalence was driven by an increase in grade 1 diarrhea; the proportion of patients with grade 2 or 3 diarrhea was generally consistent, at 6–10% in most cycles (Fig. 2G and H).

In the sorafenib group, fatigue showed the highest incidence in cycle 1 (27%); the incidence by cycle was 7% or lower in cycles 2–9. Fatigue prevalence was generally stable over cycles 1–9, fluctuating within 26–33%. Throughout the first nine cycles most fatigue was grade 1 or 2 and there was no clear shift in its severity over time (Fig. 3A and B).

Rates of new or worsening weight loss in the sorafenib group were highest during cycles 1–4. Prevalence increased during cycles 1–7, after which it stabilized (Fig. 2K and L). Weight loss was primarily grade 1 or 2. Of the AEs analyzed here, only weight loss tended to increase in severity over time, with a greater proportion of patients with grade 2 toxicity in cycle 9 compared with cycles 1 and 2 (Fig. 3C and D).

Increased serum TSH was a study-specific AE for which grade 1 was the maximum defined severity (the few reports of grade 2 increased TSH were due to errors in grading). This AE was observed throughout the study in the sorafenib group. Its incidence by cycle was low in cycle 1 (<1%), highest in cycle 2 (11%), and tended to decline thereafter. The prevalence of increased TSH was also low in cycle 1 (<1%); prevalence then rose from 12% in cycle 2 to a peak of 19% in cycle 5, after which it steadily declined to 13% by cycle 9 (Fig. 3E and F).

In the sorafenib group, hypocalcemia incidence was highest in cycle 2 (7%). The prevalence of hypocalcemia was low in cycle 1 (1%), and then generally stable at 8–11% over cycles 2–9. Most hypocalcemia was grade 1 or 2, although grade 3 and 4 events appeared early and were observed throughout the first seven cycles (grade 4) and nine cycles (grade 3) (Fig. 3G and H).

## Discussion

Detailed analysis of the AE occurrence patterns in patients with RAI-refractory DTC in DECISION demonstrated that most AEs with sorafenib were grade 1 or 2, started early during the treatment course, and were typically manageable over time. The overall rate of discontinuation of sorafenib due to AEs in DECISION was <20%, indicating that in the majority of patients with RAI-refractory DTC the drug was well tolerated, with most discontinuations due to AEs (4%) occurring in cycle 1. Similarly, the proportion of grade 2 and 3 AEs tended to be fairly stable or decline over time, with the exception of weight loss where grade 2 increased in later cycles. The persistence of AEs over the course of treatment argues for the continued surveillance and management of patients receiving sorafenib in this setting.

AEs associated with sorafenib treatment were managed with a combination of dose modifications (treatment interruptions and dose reductions) and concomitant use of other medications such as antidiarrheals, antihypertensives, or skin lotions. Although the effectiveness of these AE management methods was not quantified, the low rate of sorafenib discontinuation due to AEs beyond cycle 1 suggests that they were effective. Furthermore, the AE management profiles for individual patients over time again speak to the effectiveness of active management of AEs for patients on the DECISION trial (Supplementary Figure 2 given at the end of this article).

Some AEs, such as HFSR, alopecia, diarrhea, hypertension, squamous cell carcinoma of the skin, and hypocalcemia, were reported more frequently during the DECISION trial than in the pivotal Phase 3 trials of sorafenib in renal cell carcinoma (RCC) and hepatocellular carcinoma (HCC) (Llovet *et al*. 2008, Escudier *et al*. 2009, Brose *et al*. 2014*a*). The overall proportions of sorafenib dose interruptions (66%) and reductions (64%) in DECISION were also higher than in the Phase 3 sorafenib HCC trials (the SHARP study: 44 and 26% respectively; Asia-Pacific study: not reported and 31%) and RCC trial (TARGET study: 21 and 13% respectively) (Escudier *et al*. 2007, Llovet *et al*. 2008, Cheng *et al*. 2009, Brose *et al*. 2014*a*). Several factors could have contributed to these differences. First, the median duration of sorafenib therapy in DECISION (10.6 months) was approximately twice as long as that in TARGET and SHARP (both 5.3 months) allowing more time for events to occur and for the dose to be modified in response (Escudier *et al*. 2007, Llovet *et al*. 2008). Also, compared with TARGET and SHARP, the dose reduction scheme in DECISION allowed for a more gradual reduction in sorafenib daily dose, from 800 mg, to 600 mg, to 400 mg, to 200 mg. Thus, patients in DECISION received 600 mg/day on the first dose reduction whereas in TARGET, SHARP, and the Asia-Pacific trial doses were first reduced from 800 mg/day to 400 mg/day, and then to 400 mg every other day (TARGET and SHARP) or 200 mg/day (Asia-Pacific study) (Escudier *et al*. 2007, Llovet *et al*. 2008, Cheng *et al*. 2009).

It has been reported that AEs in patients treated with sorafenib could be related to drug exposure (Boudou-Rouquette *et al*. 2012, Pecuchet *et al*. 2012). Sorafenib exposure in DECISION patients was higher than that in patients with RCC or HCC (Bastholt *et al*. 2014), and has been reported to decrease over time in patients receiving unchanging dose (Arrondeau *et al*. 2012). However, whereas higher sorafenib concentrations have been correlated with increased rates of hypertension and grade ≥2 HFSR in other tumor types (Pecuchet *et al*. 2012), and a trend towards a higher frequency of grade 2 AEs was reported in patients with RAI-refractory DTC in patients from DECISION receiving sorafenib, no significant correlation was found between sorafenib exposure and AEs (or PFS) (Bastholt *et al*. 2014). It is possible that AEs may have been ameliorated over time due to declining drug concentrations (Brose *et al*. 2015), although longitudinal sorafenib exposure measurements were not made, and the by-cycle incidence data reported here indicate that new or worsening AEs occurred throughout cycles 1–9. The reason behind the higher AE rates in DECISION thus remains unclear (Brose *et al*. 2015).

Squamous cell carcinomas of the skin were more common in sorafenib patients treated in DECISION than in patients in the RCC and HCC pivotal studies (Llovet *et al*. 2008, Escudier *et al*. 2009, Brose *et al*. 2014*a*), and so skin cancer screening may be particularly important in this patient group (Cabanillas *et al*. 2010, Brose *et al*. 2014*a*). Cutaneous squamous cell carcinomas have previously been associated with targeted therapies that inhibit BRAF (Cabanillas *et al*. 2010). These additional primary cancers can be benign or malignant and generally respond well to timely intervention (Alam & Ratner 2001, Cabanillas *et al*. 2010, Belum *et al*. 2015).

The overall incidence of HFSR reported in the DECISION trial was higher than in the RCC and HCC trials (Brose *et al*. 2014*a*). Although the study-specific HFSR grading definitions used in the DECISION study were more inclusive than the CTCAE v3.0 definitions used in other studies (e.g. including dysesthesia and paresthesia in grade 1 HFSR) and may have contributed to the higher numbers, the 20% incidence of grade 3 HFSR exceeded previous reports, which ranged from 6 to 11% (Escudier *et al*. 2007, Llovet *et al*. 2008, Cheng *et al*. 2009). Dose modifications and treatment discontinuations in DECISION also occurred most often due to HFSR (Table 3). Dose interruptions due to HFSR (26.6%) may have been higher than those due to other AEs in part because the study protocol mandated treatment interruption for skin toxicities as low as grade 2 (if the AE was not resolved in 7 days or on second occurrence) whereas treatment interruption for other AEs was not mandated until grade 3 (with the exception of grade 2 hypertension) (Supplementary Tables 1, 2, 3 and 4 given at the end of this article). Because HFSR was the most commonly cited AE leading to treatment discontinuation (5%), and because it occurs early in treatment, prompt and effective management of HFSR would seem to be critical in maintaining patients on treatment. Most advice on the management of HFSR is empiric (Edmonds *et al*. 2012). However, there is evidence from a study in patients with HCC starting treatment with sorafenib that prophylactic use of a 10% urea-based cream can delay HFSR as well as reduce its incidence and severity (Ren *et al*. 2015). Data also suggest that in cancer patients treated with sorafenib or sunitinib, topical treatment of HFSR using a 40% urea cream in combination with a 0.1% tazarotene cream or a 5% fluorouracil cream twice daily is effective in reducing HFSR severity (Lacouture *et al*. 2008). Additionally, dose modifications in combination with symptomatic treatment have rapidly resolved symptoms of grade 3 HFSR in patients receiving sorafenib to treat RCC (Autier *et al*. 2008). HFSR prevention and management has been reviewed in detail elsewhere (Edmonds *et al*. 2012, Brose *et al*. 2014*b*, Walko & Grande 2014).

Only one patient discontinued treatment due to hypertension and no cases of serious or grade 4 hypertension, such as hypertensive crisis, were reported. Diarrhea and weight loss tended to increase in prevalence throughout cycles 1–9. An increase in grade 1 events accounted for the rise in diarrhea. A gradual increase in the prevalence of diarrhea has also been reported in RCC patients treated with sorafenib (Procopio *et al*. 2013). The severity of weight loss is defined in terms of a decrease in weight from baseline in CTCAE v3.0 (National Cancer Institute 2006), with grade 1 being a 5–10% reduction and no intervention required, and grade 2 being a 10–20% reduction with nutritional supplementation indicated. The higher proportion of grade 2 events seen in this study in later cycles may reflect the cumulative effects of continuous, gradual weight loss in some patients rather than a sign of accelerated weight loss.

TSH suppression is an important treatment intervention in metastatic DTC (Cooper *et al*. 2009, Pacini *et al*. 2012); hence, elevated TSH was recorded as a study-specific AE in the DECISION trial. TSH elevations with sorafenib were typically transient. It was anticipated that elevated TSH would occur more frequently with sorafenib than with placebo because of the known interaction between sorafenib and thyroid metabolism in athyreotic patients (Gupta-Abramson *et al*. 2008, Abdulrahman *et al*. 2010, Verloop *et al*. 2013). Indeed, elevated TSH requiring an increase in thyroid hormone replacement is considered a class effect of tyrosine kinase inhibitors (Cabanillas *et al*. 2011). Increased serum TSH was reported in one-third of sorafenib patients (Brose *et al*. 2014*a*); incidence was highest in cycle 2, and <5% thereafter. Prevalence increased gradually up to cycle 5. These results support the monthly monitoring of TSH levels and the use of thyroxine replacement medications, as appropriate (Bayer HealthCare Pharmaceuticals, Inc. 2013).

Grade 3 or 4 hypocalcemia was reported with a prevalence of 1–3% in the sorafenib group throughout the first nine treatment cycles in DECISION. There was one report of serious hypocalcemia and one patient discontinued due to hypocalcemia. The small number of transient grade 3 and 4 hypocalcemia events in the placebo group underlines the fact that hypocalcemia is a known postoperative complication of thyroidectomy (Gonçalves & Kowalski 2005, Asari *et al*. 2008) and is therefore specifically related to the patient population studied in DECISION.

These results have implications for the optimal management of RAI-refractory DTC patients receiving sorafenib. In general, AEs and the resultant dose modification, including discontinuations, tended to occur early. Once stabilized, discontinuations due to AEs were infrequent. Therefore, these results suggest that increased attention to AEs early in the course of treatment coupled with timely dose modifications may help to maximize the number of patients who can stay on therapy and potentially benefit from treatment.

## Supplementary data

This is linked to the online version of the paper at http://dx.doi.org/10.1530/ERC-15-0252.

## Figures and Tables

**Table 1:** Safety overview (safety population)

	**Sorafenib (*n*=207)**	**Placebo (*n*=209)**
Median duration of treatment, months (IQR)	10.6 (5.3–15.7)	6.5 (3.3–12.9)
Mean daily dose, mg (SD)	651 (159)	793 (26)
Dose interruptions, *n* (%)	137 (66.2)	54 (25.8)
Dose reductions, *n* (%)	133 (64.3)	19 (9.1)
Any treatment-emergent AE, *n* (%)	204 (98.6)	183 (87.6)
Grade 3/4 treatment-emergent AEs, *n* (%)	133 (64.3)	63 (30.1)
AEs leading to withdrawals, *n* (%)	39 (18.8)	8 (3.8)
Treatment-emergent deaths, *n* (%)	12 (5.8)^a^	6 (2.9)^b^
Deaths attributed to study drug, *n* (%)	1 (0.5)	1 (0.5)
Serious AEs, *n* (%)	77 (37.2)	55 (26.3)
Serious AEs reported by ≥2% of patients receiving sorafenib, *n* (%)		
Secondary malignancy	9 (4.3)	4 (1.9)
Dyspnea	7 (3.4)	6 (2.9)
Pleural effusion	6 (2.9)	4 (1.9)

AEs, adverse events; IQR, interquartile range.

a Progressive disease, 7; unknown, 2; lung infection, 1; chronic obstructive lung disease, 1; myocardial infarction, 1.

b Progressive disease, 4; pulmonary embolism, 1; subdural hematoma, 1.

**Table 2:** Overall incidence of treatment-emergent adverse events occurring in ≥10% of patients receiving sorafenib^a^ (safety population)

**Adverse event**	**Sorafenib** **(*n*=207)**, ***n*** **(%)**			**Placebo** **(*n*=209)**, ***n*** **(%)**		
Any grade	Grade 3	Grade 4	Any grade	Grade 3	Grade 4
Hand–foot skin reaction	158 (76.3)	42 (20.3)	–	20 (9.6)	0	–
Diarrhea	142 (68.6)	11 (5.3)	1 (0.5)	32 (15.3)	2 (1.0)	0
Alopecia	139 (67.1)	–	–	16 (7.7)	–	–
Rash/desquamation	104 (50.2)	10 (4.8)	0	24 (11.5)	0	0
Fatigue	103 (49.8)	11 (5.3)	1 (0.5)	53 (25.4)	3 (1.4)	0
Weight loss	97 (46.9)	12 (5.8)	–	29 (13.9)	2 (1.0)	–
Hypertension	84 (40.6)	20 (9.7)	0	26 (12.4)	5 (2.4)	0
Serum TSH increase (MedDRA)^b^	69 (33.3)	–	–	28 (13.4)	–	–
Anorexia	66 (31.9)	5 (2.4)	0	10 (4.8)	0	0
Oral mucositis (functional/symptomatic)	48 (23.2)	1 (0.5)	1 (0.5)	7 (3.3)	0	0
Pruritus	44 (21.3)	2 (1.0)	–	22 (10.5)	0	–
Nausea	43 (20.8)	0	–	24 (11.5)	0	–
Hypocalcemia	39 (18.8)	12 (5.8)	7 (3.4)	10 (4.8)	1 (0.5)	2 (1.0)
Headache	37 (17.9)	0	–	15 (7.2)	0	–
Cough	32 (15.5)	0	–	32 (15.3)	0	–
Constipation	31 (15.0)	0	0	17 (8.1)	1 (0.5)	0
Shortness of breath	30 (14.5)	10 (4.8)	0	28 (13.4)	4 (1.9)	2 (1.0)
Dry skin	30 (14.5)	1 (0.5)	–	12 (5.7)	0	–
Abdominal pain	29 (14.0)	3 (1.4)	0	8 (3.8)	1 (0.5)	0
Limb pain	28 (13.5)	1 (0.5)	0	18 (8.6)	1 (0.5)	0
ALT	26 (12.6)	5 (2.4)	1 (0.5)	9 (4.3)	0	0
Voice changes	25 (12.1)	1 (0.5)	0	6 (2.9)	0	0
Fever	23 (11.1)	2 (1.0)	1 (0.5)	10 (4.8)	0	0
Vomiting	23 (11.1)	1 (0.5)	0	12 (5.7)	0	0
AST	23 (11.1)	2 (1.0)	0	5 (2.4)	0	0
Back pain	22 (10.6)	2 (1.0)	0	22 (10.5)	2 (1.0)	1 (0.5)
Pain in throat/pharynx/larynx	21 (10.1)	0	0	8 (3.8)	0	0

ALT, alanine transaminase; AST, aspartate transaminase; MedDRA, Medical Dictionary for Regulatory Activities; TSH, thyroid stimulating hormone.

a Nonspecific AEs not included in this table: dermatology – other, metabolic/laboratory – other, and pain – other.

b Study-specific AE including TSH concentrations >0.5 mIU/l. Maximum possible severity was grade 1.

**Table 3:** Study drug interruptions, reductions, and permanent discontinuations due to specific adverse events over the entire course of treatment (safety population)

**Adverse event**	**Sorafenib group** **(*n*=207)**, *n* (%)			**Placebo group** **(*n*=209)**, *n* (%)		
Interruption	Reduction	Discontinuation	Interruption	Reduction	Discontinuation
Hand–foot skin reaction	55 (26.6)	70 (33.8)	11 (5.3)	0	2 (1.0)	0
Rash/desquamation	18 (8.7)	16 (7.7)	3 (1.4)	0	0	0
Hypertension	16 (7.7)	12 (5.8)	1 (0.5)	3 (1.4)	1 (0.5)	0
Diarrhea	7 (3.4)	28 (13.5)	2 (1.0)	2 (1.0)	1 (0.5)	0
Fatigue	15 (7.2)	7 (3.4)	3 (1.4)	3 (1.4)	3 (1.4)	0
Weight loss	5 (2.4)	13 (6.3)	1 (0.5)	2 (1.0)	1 (0.5)	2 (1.0)
Hypocalcemia	4 (1.9)	6 (2.9)	1 (0.5)	0	0	0
